# Robust vehicle detection in different weather conditions: Using MIPM

**DOI:** 10.1371/journal.pone.0191355

**Published:** 2018-03-07

**Authors:** Nastaran Yaghoobi Ershadi, José Manuel Menéndez, David Jiménez

**Affiliations:** E.T.S. de Ingenieros de Telecomunicación, Universidad Politécnica de Madrid, Madrid, Spain; Mar Ephraem College of Engineering & Technology, INDIA

## Abstract

Intelligent Transportation Systems (ITS) allow us to have high quality traffic information to reduce the risk of potentially critical situations. Conventional image-based traffic detection methods have difficulties acquiring good images due to perspective and background noise, poor lighting and weather conditions. In this paper, we propose a new method to accurately segment and track vehicles. After removing perspective using Modified Inverse Perspective Mapping (MIPM), Hough transform is applied to extract road lines and lanes. Then, Gaussian Mixture Models (GMM) are used to segment moving objects and to tackle car shadow effects, we apply a chromacity-based strategy. Finally, performance is evaluated through three different video benchmarks: own recorded videos in Madrid and Tehran (with different weather conditions at urban and interurban areas); and two well-known public datasets (KITTI and DETRAC). Our results indicate that the proposed algorithms are robust, and more accurate compared to others, especially when facing occlusions, lighting variations and weather conditions.

## Introduction

Recently, accurate and real-time traffic information detection in different weather conditions has become a significant problem. Early researchers have attempted to use it in different traffic related applications, such as traffic management, traffic control, decision-making, and vehicle scheduling. Practical and useful traffic related information includes, but is not limited to, traffic volume/stream, speed of vehicles, detecting/locating accidents, movements between lanes, or the distance between consecutive vehicles. Given the fact that there are different types of vehicles with different speeds and behavoiur, various approaches have been proposed and applied to gather such wide traffic related information. So far, ultrasonic detection methods, electromagnetic induction-based devices, as well as video-based traffic approaches, have been used. One of the earliest methods was ultrasonic sensor-based devices, Kim et al. [1] explain that although they seem economically efficient, their data collection capability is limited as only averaged vehicle speed and/or number of passing vehicles in a certain period can be obtained by ultrasonic based devices. Furthermore, the authors concluded that a high-speed weight-in-motion (HSWIM) equipment which uses loop/piezo sensors is able to obtain comprehensive traffic data such as speed, length, occupancy, axle weight, and vehicle category. However, the main disadvantage of such systems is their relatively high cost and difficult sensor installation, as they need to be buried under the pavement.

Video-based traffic detection methods, on the other hand, are fairly cost efficient, simple, and more importantly, thanks to recent technological developments, widely available. Vision based methods seem to be highly promising as they are not only independent of reconstruction of pavement, but they also provide more potential advantages including more flexibility compared to inductive loops, as well as larger detection areas. Investigation of vehicle detection methods based on video camera began in the late 1970s. Naveen et al. [2] proposed a video-based method to detect the vehicles with Harris-Stephen corner detector algorithm. The algorithm was used to develop a vehicle detection and tracking system to eliminate the need for complex setting, robustness to different variations but it just works with low-resolution videos and they do not test the method under bad weather conditions. However, using video-based detection approaches raises interesting yet difficult problems in the field of image processing. For instance, robust detection algorithms are required as light conditions vary dramatically throughout seasons, weeks, and even in one single day. To overcome such problems, a large amount of computational burden is imposed on the system, which mostly impedes the application of video-based algorithms in real-time traffic monitoring systems.

## State of the art

In the literature, there have been several researchers in the field of vision-based traffic information detection. In 1994 Yuan [3] used a single perspective image taken by a camera at the roadside, proposed a method to detect vehicles and estimate their length, width, height as well as the total number of vehicles. However, his approach was based on remapping images using homogeneous methods, which reduced accuracy results. One of the approaches to eliminate the negative effects of the perspective is to use Inverse Perspective Mapping (IPM). This approach was originally introduced by Mallot [4]. However, Bertozzi et al. [5] reported that IPM re-sampled non-homogeneously in order to produce a new image that represents the same scene as acquired from a different position. Muad et al. [6] used IPM for lane detection task and vehicle navigation development. The Image quality is not good after IPM transformation [4,6]. Chien et al. [7] presented the Top-View Transformation Model for image coordinate transformation, TVTM transforming a perspective projection image into its corresponding bird’s eye vision. However, they did not test the method under public datasets and in different lighting and weather conditions. Wang et al. [8] detected traffic stream and volume using an algorithm based on inverse perspective mapping (IPM). They used IPM to eliminate the geometric distortion related to image sequence. In addition, marking lines in the lane area were extracted by introducing geometric constraints of the road structure. Furthermore, using a background difference method, they extracted the vehicle sequence contours, and, accordingly, measured traffic stream, they presented two different types of metrics which were the vehicles contour area based method and the vehicles queue length based method. However their system is not suitable for all types of roads and all traffic conditions. Daiming et al. [9] proposed an automatic inverse perspective mapping method based on vanishing point, which is adaptive to the uphill and downhill road even with slight rotation of the main road direction. Vanishing point detection and the inverse perspective mapping process for each frame, results in high computational complexity. In [10] the authors presented a system (hardware and software) for lane and obstacle detection, satisfying the hard real-time constraints imposed by the automotive field. The main innovative contribution of this work is used in the IPM technique to simplify both low and medium level processing steps. Jiang et al. [11] used fast inverse perspective mapping algorithm (FIPMA) to reduce the computational expense of IPM, but its performance was changed by the effect of the video quality. They also used the gradient operator to extract edge information of lane markings, such as magnitude and orientation. However, we use Hough transform, which is considered a powerful tool in edge linking for line extraction, and it is quite insensitive to noise, which is a very good strategy when the video is captured under varying whether conditions. Lin et al. [12] developed a vision based obstacle detection system by utilizing a proposed fisheye lens inverse perspective mapping (FLIPM) method. The new mapping equations were derived to transform the images captured by the fisheye lens camera into the undistorted remapped ones under practical circumstances. Regarding obstacle detection, they made use of the features of vertical edges on objects from remapped images to indicate the relative positions of obstacles. The static information of the remapped images in the current frame determined the features of source images in the searching stage from either the profile or temporal IPM difference image.

However, it should be emphasized that the performance of these methods can be exacerbated by the perspective and the geometric properties of the objects in an image which may have been distorted by different lighting and weather conditions. Such distortions reduce the accuracy of the measurement and, in turn, the performance and accuracy of the traffic information detection algorithms.

Vehicle detection and counting play an important role for estimating traffic flow. Yingjie et al. [13] proposed virtual loop method to improve the quality of video-based vehicle counting.Their application does not perform any training activity or model building, and it does not adapt itself to changing scenarios. Counting activity is presented on single or multiple lanes, where virtual loops are manually placed and all vehicles are moving in the same direction. Andrea in [14] described a street viewer system for traffic behavior in different scenarios, the systems accuracy in sunny weather is about 90%, cloudy weather 98.23% and rainy weather 84.91%. Results indicated that our method accuracy rates are higher than those. Grzegorz et al. [15] proposed a method to detect vehicles that stop in restricted areas, the proposed algorithm uses the background subtraction results for detection of moving objects, and then pixels belonging to moving objects are tested for stability. Hence, detection of stationary objects which were previously moving is possible and if the object has stopped in a designated area, the event is declared. The accuracy of the proposed method is 76.9% in a real world scenario. Pawel et al. [16] proposed an algorithm for the analysis of a moving vehicles trajectory using vision-based techniques. They used background modelling, object tracking, and homographic projection. This paper integrated the information about the movement of vehicles obtained from more than one camera. They included a shadow detection and elimination method, assuming that casted shadows lower the luminance of the point while chrominance is unchanged. The valid color space for their method was HSV. As they mentioned, the system was a good deterrent from dangerous and illegal driving behavior, and contributed to safety protection and fluent traffic flow. However, they did not test the accuracy of the method in images with different lighting conditions.

Indrabayu et al. [17] proposed a method for Vehicle Detection and Tracking using Gaussian Mixture Model and Kalman Filter. They used a dataset under two different conditions, light traffic and heavy traffic. The detection accuracy for their method in light traffic conditions was 97.22%, and in high traffic was 79.63%. Data collection was only done during the day, which provided limited results. They neglected to include data from harsh weather conditions and poor lighting. Mohammad et al. [18] presented a system for a vehicle's traffic behavior. They used Gaussian mixture model for each frame to achieve a precise background image.

The received images analyzed along with the trend images to extract the vehicles. Then, a green block surrounded each vehicles to enable the researches to count them. In the 4^th^ phase, the optical flow was used for computing moment velocity of each vehicle based on improved Lucas Kanade and Horn-Schunck methods. Accuracy of their method was 97.19% under normal weather conditions in 8 different places in Shiraz. (We computed the average results of different places). We have compared the results of methods in [17] and [18] with our method to verify that our approach has the highest accuracy, even under different conditions. In conclusion, in order to develop better intelligent transportation systems (ITS), it is essential to present a method that is not only able to eliminate the perspective from the images, but is also capable of producing an image from the original image, from which real traffic information can be easily and accurately extracted.

In this paper, we propose a vision based, real-time traffic information detection algorithm that uses modified inverse perspective mapping MIPM. This method is recommended to remove the perspective from images to accurately detect vehicles in various weather conditions. Our simulation results verified the better performance of the proposed method compared to similar works in delectability and traceability under different weather conditions, perspective and background noise, shadows and lighting transitions, all of which are difficulties conventional traffic detection methods have to deal with. As indicated in the experimental results section, our proposed method performs under these challenging conditions better than those traditional approaches.

## Proposed enhancement

In order to achieve our goals in this work, first we eliminated the perspective from the images using a newly proposed Modified Inverse Perspective Mapping (MIPM); afterward, using Hough transform [19], we extracted structural information, such as road lines and lanes; then, a binary image was produced using a Gaussian Mixture model [20], in a way that the road and the moving vehicles were displayed in white and black colors respectively. As we have to obtain the car area, shadows must be removed, but when using Gaussian Mixture Models, shadows are usually combined with the car area. This is caused by the fact that shadows share the same movement patterns as the vehicle; moreover, shadows demonstrate a similar magnitude of change in intensity as those of the foreground objects. To overcome this issue, we used the Chromacity-based method [21]. Finally, we extracted the required traffic information, such as movement speed of vehicles, area of vehicles (used for classification purposes), types of movement with respect to the structural information of the road and the distance between vehicles. The proposed procedure has been tested with our datasets and two public data sets that contain normal, rainy and snowy weather conditions, different lighting conditions (sunny and poor lighting) and different types of locations (urban, interurban, intersections, highway, etc.). The results show that our strategy has significant effect in occlusion and in complex sequences and conditions. This paper is organized as follows: next section provides the details to extract real traffic information through different techniques like Modified Inverse Perspective Mapping, Hough transform and Gaussian Mixture Models to detect the vehicle. Following, the data set and experimental results of the proposed algorithm are presented. Next section deals with the comparison of the different methods and validation. Finally, conclusions and future work are presented in the last section.

## Detailed description to extract real traffic information

**Fig 1:**
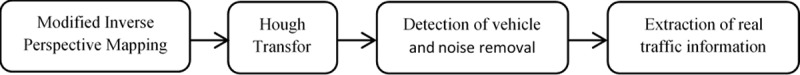
Global description of extract real traffic information.

The general structure of the proposed traffic information detection algorithm is illustrated in “Fig 1”.

### Description of modified inverse perspective mapping

Obtaining information about the surrounding environment is a crucial task for biological organisms as well as artificial systems designed for autonomous navigation or driver assistance applications. Inside the camera or the eye, however, on the image plane where the 3D scene is projected, the effects of perspective will complicate most high level information retrieval problems [22]. Inverse perspective mapping (IPM) is a geometrical transformation of the family of re-sampling filters; the initial image is non-homogeneously re-sampled to produce a new image that represents the same scene as acquired from a different position [5].

#### Removing the perspective effect

IPM method is capable of removing the effect of perspective from the initial image. However, IPM affects the geometric properties of subjects in the newly produced image as it produces a non-homogeneous image. By non-homogeneous, we mean the image is not regular, the environment and the car area are not easy to analyze accordingly. Any two images of the same planar surface in space are related by a homography, the homography transfers points from one view to the other. The distortion increases as the distance and angle are increased [23]. In this paper, considering the distance between the vehicles and the camera, we proposed the use of a weighting factor, which is related to longitudinal and lateral direction. This way, the classical perspective distortion is reduced, and the detection ability and traceability of the vehicles are maximized using simple but effective image processing strategies. This is one of the comparative advantages of the proposed method against IPM and Homography method.

#### I -> S mapping

**Fig 2:**
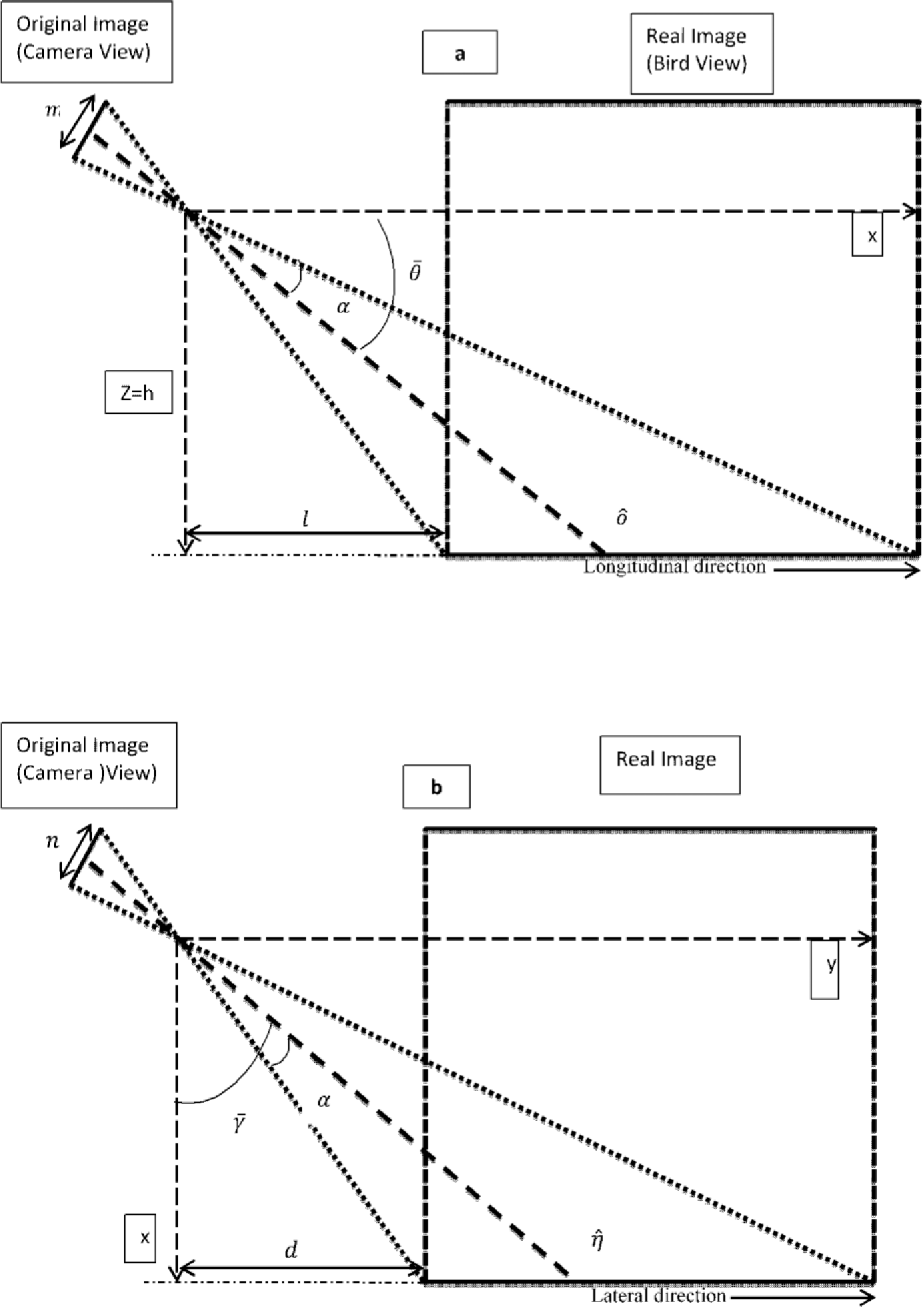
a) The *zx* plane b) The *xy* plane in the *W* space, namely the *S* surface.

In order to be able to use MIPM transform, one would require the knowledge of the following parameters [5]:

- W = {(x,y,z)}∈E^3^, Which represents the real world in a three-dimensional space (world-coordinate system).
- I = {(u,v)}∈E^2^, which represents the two-dimensional image space (image-coordinate system), which is obtained by projection of the originally three dimensional scene. The I space corresponds to the image taken by the camera, while, considering the flatness of the image, the remapped image is defined as the *xy* plane of the *W* space, namely the *S*≜{(*x*,*y*,0)∈*W*} surface.
- *E*^3^ and *E*^2^ are respectively 3-dimensional (3D) 2-dimensional (2D) Euclidean space.
- Each pixel of the remapped image {(*x*,*y*,0)∈*W*} assigned to (*u*(*x*,*y*,0),*v*(*x*,*y*,0))∈*I*
- Viewpoint: position of the camera *C* = (*l*,*d*,*h*)∈*W*
- Viewing direction: the optical axis *ô* defined by the angles below.
- *γ̅*: The angle which is formed by the projection (defined by *η^*) of the optical axis *ô* on the plane z = 0 and the axis x, as illustrated in “Fig 2B”.

- *θ̅*: The angle formed between the optical axis *ô* and x axis, as depicted in “Fig 2A”.
- Aperture: the camera angular aperture which is 2α.
- Resolution: the camera resolution which is *m*×*n*.

In this paper, to get the mapping from I space to S surface, we used MIPM as formulated in Eq 1:

$$\{\begin{array}{c} x\left(u,v\right)=h\times cot\left[\left(\bar{\theta }-\alpha \right)+\frac{u\times 2\times \alpha }{m}\right]-l\\ y\left(u,v\right)=x\times \tan\left[\left(\bar{\gamma }-\alpha \right)+\frac{v\times 2\times \alpha }{n}\right]-d\\ z\left(u,v\right)=0 \end{array}.$$ (1)

#### S -> I mapping

**Fig 3:**
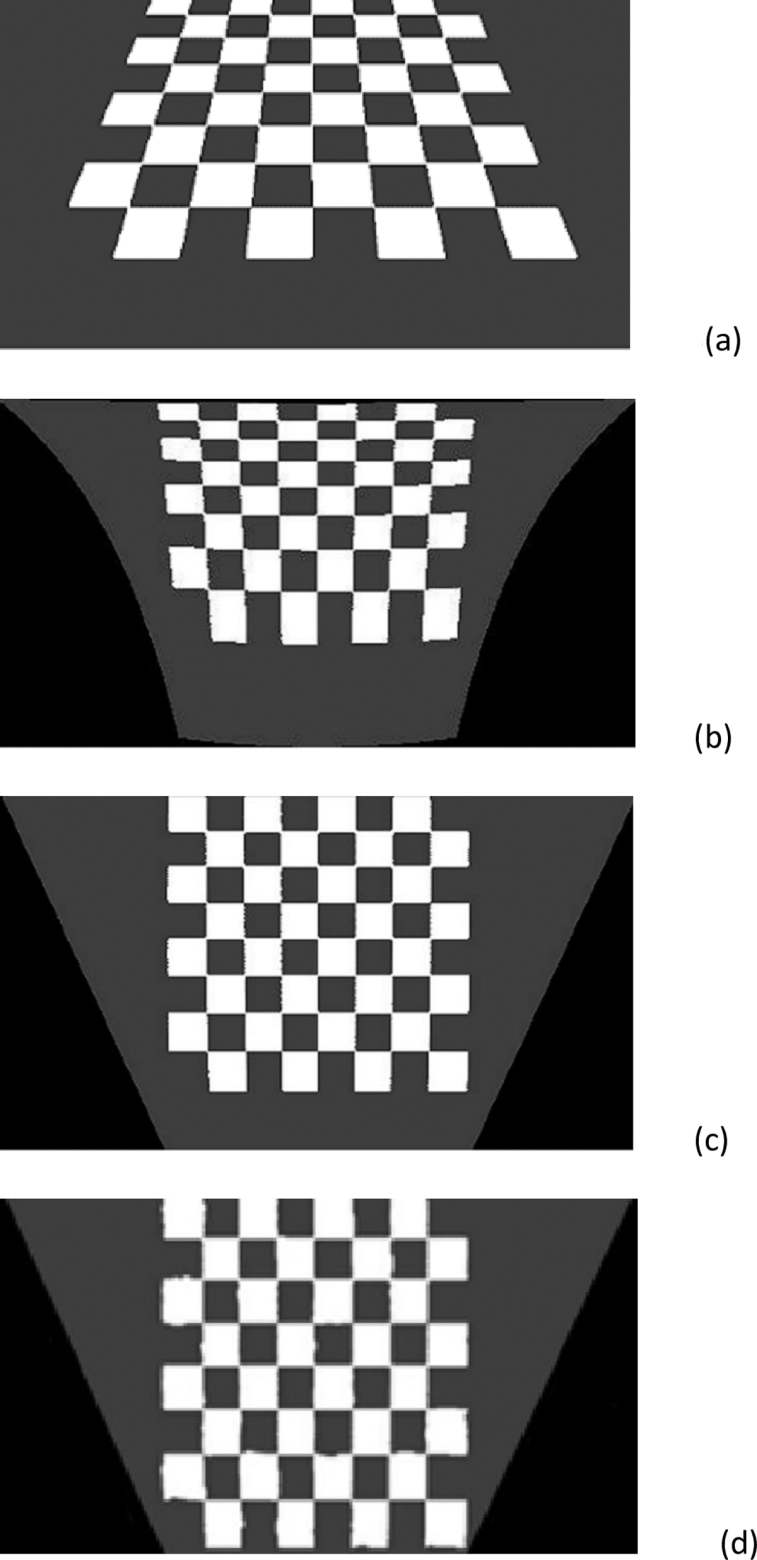
Differences between IPM, Homography and MIPM a) Original image b) IPM method c) MIPM method d) Homography method.

Using Eq 2, u and v are obtained in I surface, and as we see in “Fig 3” and section “Inherent geometry characteristic”Images obtained by MIPM are so much clearer than images by IPM or homography methods, this clearness means it can be easily used in detection, computations and calculation of the car area.

$$\{\begin{array}{c} u\left(x,y\right)=\frac{m}{2\times a}\times \left(arccot\left[\frac{x+l}{h}\right]-\bar{\theta }+a\right)\\ v\left(x,y\right)=\frac{n}{2\times a}\times (\arctan\left[\frac{y+d}{x}\right]-\bar{\gamma }+\alpha ) \end{array}.$$ (2)

Although both IPM and MIPM remove the perspective effect, in section “Inherent geometry characteristic” we see that MIPM shows more homogeneous surface than IPM, and we can easily analyze for subsequent calculations. In the real world, cars are 3D, but to compare IPM and MIPM, our assessment is in 2D. The difference between the proposed MIPM and the original IPM can be simply demonstrated in Fig 3. The selected camera angle with the horizontal axis is 45 degrees, since good results can be obtained by this angle. Also we compared MIPM to the homography method, as shown in “Fig 3D”. The result, applying homography is not as clear as MIPM. Different frames in different locations, weather conditions, lighting conditions and traffic volume were tested and are presented in this paper.Section “Data set and testing results“.

#### Comparative advantages of the proposed method against IPM and homography

- The perspective distortion is reduced, and the ability to detect and trace the objects is maximized using simple but effective image processing strategies.
- The image obtained by MIPM is much clearer than those obtained by IPM and the homography method; this clearness means it can be easily used in computations and calculation of actual geometrical measurements, such as the car area.
- In section “Inherent geometry characteristic”, we see that MIPM shows more homogeneous surface than IPM, and we can easily analyze for subsequent calculations.

When using MIPM to remap images, geometric features of the road can be easier and more efficiently extracted from the remapped picture compared to the IPM version. (section “Inherent geometry characteristic”).

### Description of Hough transform

The Hough transform is commonly used in image processing, analysis and machine learning and in recognizing general shapes as well as geometric known curves, among them, straight lines. This is done by determining local patterns, ideally a point (maximal accumulation), in a transformed parameter space [24]. (Details of the implementation are provided in section “Locating the lane area with Hough transform”).

Much of the efficiency of the Hough transform relies on the quality of the input data [25]. The edges must be detected well for the Hough transform to be efficient [25].

Use of the Hough transform on noisy images is a very delicate matter and generally, a denoising stage must be used beforehand. In the case the image is corrupted by speckle, such as working with radar or dusty images, our method performs better when detecting lines, because it attenuates the noise through summation.

According to Hough Transform every single pixel in an images space corresponds to a line inside a parameter space. The Hough transform uses *r* = *x*.cos *θ* + y.sin *θ*. Which can be rewritten $y=-\frac{\cos\;\theta }{\sin\;\theta }.x+\frac{r}{\sin\;\theta }.$ The Parameter θ Theta (radians) is the angle of the line with the range of $-\frac{\pi }{2}\leq \theta <+\frac{\pi }{2}$ and indicates the spacing of Hough transform bins along the theta-axis. The Parameter r Rho (pixels) is the distance from the line to the origin and indicates the spacing of the Hough transform bins along the rho-axis.

We used a relative threshold to extract the unique (r, θ) points relevant to each of the straight lines in our original image.

We selected threshold for local maxima = 75 and for Gap Allowed (Number of Pixels) = 50. Thinning is a morphological operation. It used to remove selected foreground pixels from binary images. The thinning here is an operation related to the hit-and-miss transform. The thinning of an image I by a structuring element J is *thin* **(***I*,*J***) =** *I***–***hit***–***and–miss***(***I*,*J***).** The subtraction is a logical subtraction defined by *X*–*Y=X*∩*NOT* Y.

### Detection of foreground using Gaussian mixture models

Detection of moving objects is an interesting field of research. In many vision systems, namely video surveillance and more importantly traffic monitoring, capability of extracting moving objects using a sequence of video is highly crucial and fundamental. To successfully track moving objects, analyze movements of patterns, or classify interested objects, it is of utmost importance to reliably perform movement detection.

#### Foreground detection methods

Moving objects detection methods can be categorized into three main groups [26]: (i) temporal differencing [27], (ii) optical flow block based obstacle detection [28], and (iii) background subtraction [29,30]. Although temporal differencing can easily adapt to dynamic environments, it is known for the demonstration of poor performance especially in extracting all relevant feature pixels. On the other hand, optical flow is able to detect moving objects while the camera is moving. However, the majority of optical flow methods cannot be used in full-frame video streams in real-time applications, unless specialized high speed hardware is available, because they impose high computational burden on the system. So far, background subtraction has proven to be the category applied most successfully in practice as they can provide the most complete feature data. The basic idea of background subtraction methods is to estimate the background and evolve its estimation frame by frame; then, it uses the differences between the current frame and the current background model to detect moving objects. However, it should be noted that it is highly sensitive to dynamic scene changes caused by lighting and other extraneous events like bad conditioned weather.

#### Implementation of Gaussian mixture model (GMM)

In the field of image processing, a lot of different research has been carried out, with the main purpose of presenting efficient and reliable background subtraction. Considering the statistical features applied to constructing the background model, most of the methods proposed in this field can be categorized into methods based on minimum and maximum values, median value, single Gaussian, multiple Gaussians [31] (also known as Gaussian Mixture model (GMM)), etc. Among subtraction methods, GMM is known to be the most accurate approximation for processing practical pixels. One single adaptive Gaussian per pixel should be enough, as long as each one of the pixels results from a single surface with lightings that are fixed or slowly changing. Each pixel’s Gaussian is updated over time. However, practically speaking, such conditions do not hold in frames as multiple surfaces often appear in a particular pixel and the lighting of the frames changes. Consequently, single Gaussian methods cannot be used and GMM, which is required for detecting the model of the background. In all the methods based on GMM for background modeling, each pixel of the frame is modeled by generally 3 to 5 Gaussians. Each one of Gaussians indicates the expectation that samples of the same scene point are likely to display Gaussian noise distribution function. On the other hand, multiple Gaussians indicate that more than one process type may be detected over a period of time. Applying multiple Gaussians used to impose high computational burden on the system. However, this is not the case nowadays, since researchers have proposed several simplifications to reduce computational complexity which makes them suitable for real-time applications [31]. Moreover, multiple Gaussian approaches are desirable methods as they require much less storage capacity due to the fact that, unlike other classes of methods (such as median value methods), they do not need to store numerous preceding frames. GMM based methods are able to successfully handle gradual lighting changes as they slowly adjust parameters of the Gaussians [32,33]. Additionally, GMM based methods are also capable of handling multimodal distributions caused by real world application issues such as shadows, swaying branches, secularities, computer monitors, which are generally ignored in computer vision literature. For example, holes created in objects or still left in that stop moving are taken into account as the background model, which benefits subsequent detection. Moreover, when the background reappears in the image, GMM responds fast and recovers quickly. Finally, GMM automatically creates a pixel-wise threshold which is generated to flag potential points as moving object. In GMM, every pixel in a frame is modelled into Gaussian distribution. First, every pixel is divided by its intensity in RGB, then every pixel is computed for its probability, and included in the Foreground and Background.

$$P\left(X_{t}\right)=\sum_{i=1}^{K}\omega_{i,t}\;\eta \left(X_{t}\mu_{i,t},\sum_{i,t}\right).$$ (3)

X_t_ is the current pixel in frame I_t_, and t represents time.

The parameter K is the number of Gaussian. Stauffer and Grimson [34] proposed to set K from 3 to 5. In inclement weather, which include snow, wind and rain we used K = 4 and 5 to control the movement of snow, blowing leaves, and so on. Additionally, the experimental result indicate that if K is 5 in situations involving slow speed or pause of the vehicles (because of the heavy snow), the extracted foreground regions are more clear.

*ω*_*i*,*t*_ is a weight associated to the ith Gaussian at time t with mean μ_*i*,*t*_ and Σ*i*,*t* is the covariance matrix of the ith Gaussian in the mixture at time t. η is a Gaussian probability density function than we have

$$\eta \left(X_{t}\mu_{i,t},\sum_{i,t}\right)=\frac{1}{{\left(2\pi \right)}^{\frac{n}{2}}{\left|\sum_{i,t}\right|}^{\frac{1}{2}}}e^{-\frac{1}{2}\left(X_{t}-\mu_{i,t}\right)\sum_{i,t}^{-1}(X_{t}-\mu_{i,t})}.$$ (4)

Than the covariance matrix is [34]

$$\sum_{i,t}=\sigma_{i,t}^{2}I.$$ (5)

The Background is classified for every Gaussian that is bigger than the designated threshold, and the foreground is classified for the other distribution that is not included in the previous category. The first B Gaussian distributions which exceed certain threshold T (a fraction between background and foreground distribution) are retained for a background distribution.

Note that T is based on the background scene and the number of components in the Gaussian mixture model. In this paper we obtained it from a testing procedure, that is T = 0.78.

T = 0.1 leads to the situation that all background distribution is not covered and T = 0.9 lead to a situation in which the foreground distribution is combined with the background distribution.

$$B={\mathrm{argmin}}_{b}(\sum_{i=1}^{b}\omega_{i,t}>T).$$ (6)

The value *ω*, μ, σ is updated if a pixel matches with one of the K Gaussian.

$$\omega_{i,t+1}=\left(1-\propto \right)\omega_{i,t}+\propto .$$ (7)

$$\mu_{i,t+1}=\left(1-\rho \right)\mu_{i,t}+\rho .X_{t+1}.$$ (8)

$$\sigma_{i,t+1}^{2}=\left(1-\rho \right)\sigma_{i,t}^{2}+\rho \left(X_{t+1}-\mu_{i,t+1}\right).{\left(X_{t+1}-\mu_{i,t+1}\right)}^{T}.$$ (9)

Where α the predefined learning rate and ρ is the calculated learning rate. A slowly changing background needs a small learning rate, a fast changing background needs a larger learning rate. We used α = 0.1

$$\rho =\alpha \times \eta (X_{t+1},\mu_{i},\Sigma i).$$ (10)

If every parameter has been found, then the foreground detection can be performed.

$$\omega_{j,t+1}=\left(1-\alpha \right)\omega_{j+t}.$$ (11)

If all K Gaussian do not match, the pixel is classified as foreground. A binary mask is obtained, then, to make the next foreground detection, the parameters must be updated. Once the parameter maintenance is done, foreground detection can be completed and so on.

In our proposed method, mean and standard deviation are initial values, which are affected by extraction of foreground regions. We got the best results for our complex traffic scene with mean value = 349 and standard deviation = 100.

**Fig 4:**
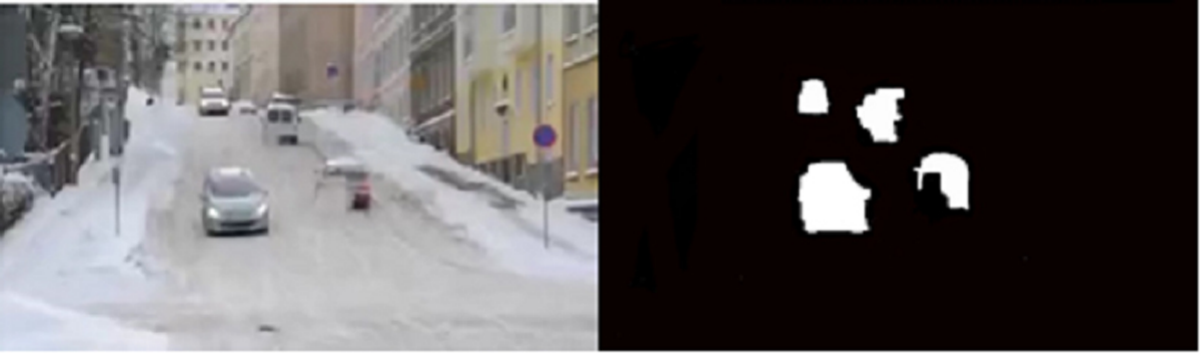
An example of video sequences with background subtraction result (Snowy weather with strong wind).

There are some noisy pixels in the foreground objects, so the basic operation of mathematical morphology is used to constitute a morphological filter to eliminate the noisy pixels in foreground and reduce disturbance. The sample result from background subtraction in snowy weather condition is illustrated in “Fig 4”.

#### Chromaticity-based method for shadow detection (HSI color space)

Generally speaking, Gaussian Mixture models suffer from a major disadvantage: shadows sometimes are detected as part of the foreground. This phenomenon is caused by the fact that shadows demonstrate movement patterns that are the same as the main moving object and also represent a magnitude of intensity change similar to the foreground objects. To overcome this issue, several methods have been proposed in the literature such as Chromaticity-based methods [21], Geometry-based methods [35], Physical methods [36], Large region (LR) texture-based methods [37], and Small region (SR) texture-based methods [38]. In order to have successful application of chromaticity methods it is of utmost importance to choose a color space with a separation of intensity and chromaticity. It has been proven that in order to have a robust shadow detection algorithm, some color spaces are suitable, such as HSI, c1c2c3 and normalized RGB. In this study, we have chosen the HSI approach. This selection provides a natural separation between luminosity and chromaticity for our proposed method, and leads to better detect ability of our method. (section “Removing of shadows in HSI space”). It should be noted that Cucchiara-based shadow detection approach has been successfully applied in surveillance applications [39, 40]. As mentioned earlier, value (I) is a measure to quantify the intensity; thus, values of (I) related to pixels, in the shadowed part, should be lower than those of the pixels in the background. Following the chromaticity cues, a shadow on the background does not show a change in its hue (H). It should be mentioned that the authors observed that if the shadow was cast on a point, the saturation (S) of the point would decrease. To sum up, we suggest that a pixel p should be detected as a part of a shadow if the following three conditions are satisfied:

β1≤(FpIBpI)≤β2. (12)

$$\left(F_{p}^{s}-B_{p}^{s}\right)\leq \tau s.$$ (13)

$$\left|F_{p}^{H}-B_{p}^{H}\right|\leq \tau H.$$ (14)

Where *F* and *B* represent the component values of HSI for the pixel position *p* in the frame (*F*) and in the background reference image (*B*), respectively. *β*_1_,*β*_2_,τ*s* and τ*H* represent thresholds that were optimized empirically.

We have tested multiple values with several thresholds, to obtain the best results in all the tested sequences with values around the following ones:

$$\beta_{1}=0.31,\;\beta_{2}=0.57,\;\tau H=0.1,\;\tau H=0.5$$

## Data set and testing results

We performed all of our experiments on a desktop PC operating Windows 8.1 with a 2.50 GHz Intel® Core™ i7 CPU 16-GB RAM and 64-bit operating system. We used MATLAB 2014 for the simulation. Our method can process around 10 frames per second.

The used datasets are representative of the problem that we have tackled because they include images in which the extraction of information for ITS applications is problematic, such as occlusions due to perspective, noise (snow, rain, fog, etc.), shadows, lighting reflections due to car lights, etc.

We have used three datasets: the first one captured in Madrid and Tehran by us, the other two are the well-known public datasets KITTI and DETRAC (More explanations and examples of sequences are provided below).

**Fig 5:**
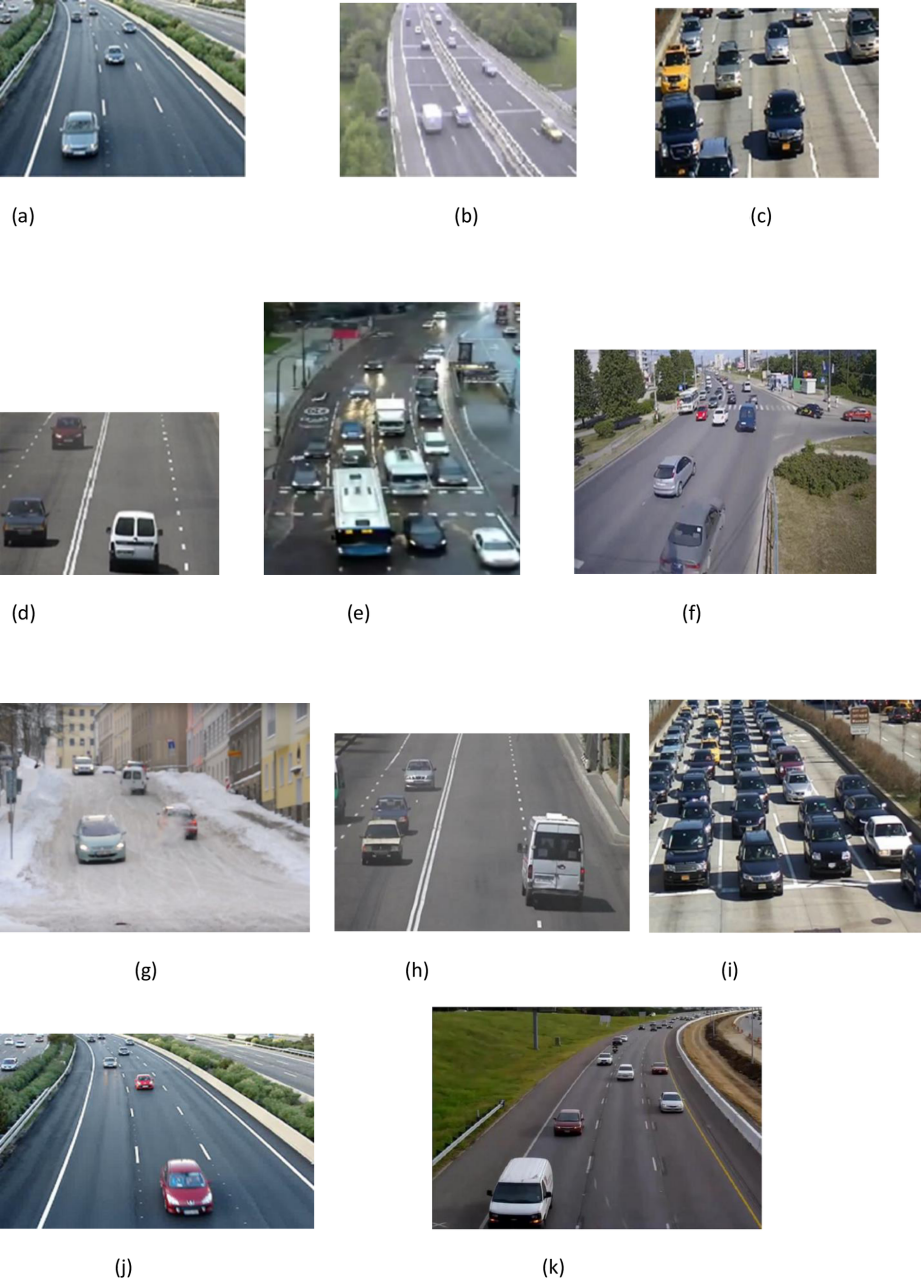
Different frames in different locations used for testing purposes (Recorded in Madrid and Tehran) (a- k) a, b, c, d) Explanation in the text, e) Rainy weather with poor lighting, wet ground, high traffic and occlusion. urban location with camera placed in the center at 45 degrees, (f) City intersection, Camera is located on right side of the street at a 45 degrees angle, in day traffic, (g) Snowy weather, urban area with low traffic, Camera angle is 45 degrees, center of an uphill street, (h) Normal weather condition, highway with low Traffic, camera angle is 30 degrees, right side of the highway Urban area, (i) Sunny weather condition, highway with high Traffic, camera angle is 45 degrees and Center of the highway, (j) Sunny weather with low traffic, camera angle is 45 degrees. Center of high way, interurban area, (k) Sunny weather with low traffic, camera angle is 45 degrees. Center of highway.

For a fair evaluation of our proposed method we tested it with different datasets that were captured in Madrid and Tehran over a period of more than six days in different locations (highway, urban road, interurban, intersection), different weather conditions (normal, snowy, rainy), different occlusions between cars, different lighting conditions (sunny, poor lighting condition and cloudy) and different traffic volume (day traffic, high traffic). They also include different types of vehicles (city cars, buses, vans, minivans, pickup trucks, etc.). The acquisition frame rate is 30 frames per second. We collected more than 80 videos, some of them up to 5 minutes in length. These videos contain up to 2500 frames. Our system is able to monitor an area of, approximately, 80 × 12 meters of the road. The frame resolution is 720 × 576. “Fig 5” show examples of our sequences with different conditions. Note that the detection rate on video is computed in a ground-truth way, we labeled the vehicles manually in selected frames, then we compared the detection result and the labeled data of these frames to compute the detection rate as Success Rate. We randomly selected sequences in different locations and conditions to compare our method with other methods. In “Fig 5” first row (left) depicts cloudy weather with a camera angled at 45 degrees, the camera is located in the center of the bridge on an urban highway; first row (right) shows normal weather conditions with a camera angled at 45 degrees in an interurban area. The camera location is on the left side of the highway on the bridge. 2^nd^ row (left) shows an urban area with sunny weather and a camera angled at 30 degrees, the location of the camera is in the center of the bridge. 2^nd^ row (right) corresponds to an urban area with a camera angled 20 degrees from the ground, located on the right side of the bridge. The remaining explanations are below each figure. Description and compared result with other methods are included in section “Comparison of the different methods and validation”.

### KITTI data set

**Fig 6:**
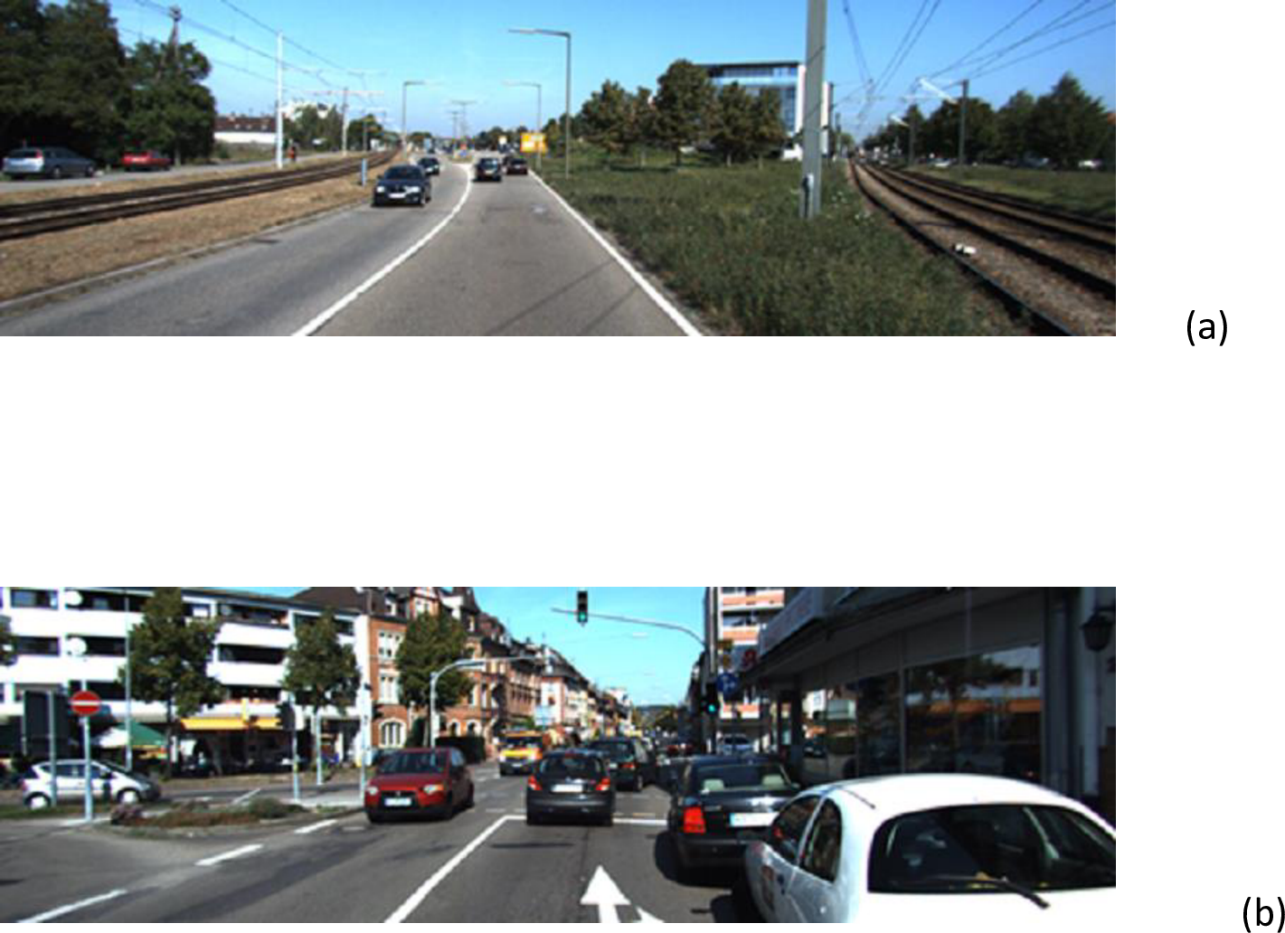
Example of sequences in KITTI Data set (a-b).

Additionally, we tested our MIPM, IPM and Homography methods with the KITTI data set [41]. We selected 150 frames with resolution 375 × 1242 pixel. The recordings in KITTI are from five different days. All datasets are color stereo images in good (sunny) weather conditions and shadowed conditions. The selected frames are in different categories of the KITTI-ROAD dataset (urban, urban two-way road, urban multi-lane road and contain different types of cars, vans, buses, etc…). “Fig 6” shows examples of randomly selected sequences. Our experiment results and explanation can be find in section “Comparison of detection rate using MIPM, IPM and Homography methods in public datasets”.

### DETRAC data set

**Fig 7:**
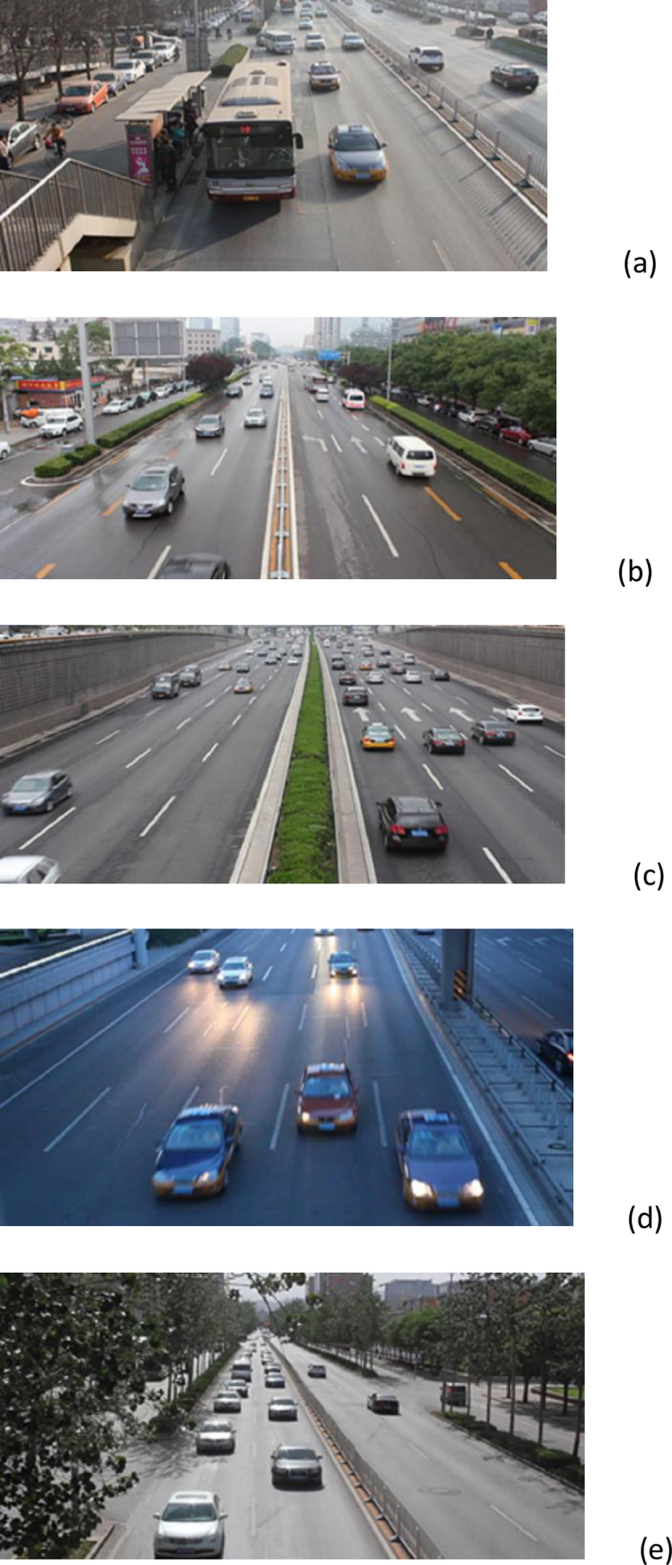
Example of sequences in DETRAC dataset (a-e).

The data set [42] consists of 10 hours of videos captured with a Cannon EOS 550D camera at 24 different locations in China. The videos are recorded at 25 frames per seconds (fps), with resolution of 960×540 pixels. Vehicle types are car, bus, van and others. We selected 150 sequences (50 sequences for each group) with different lighting conditions and occlusion, day traffic, high traffic, intersection, urban and interurban. “Fig 7” shows examples of selected frames. Explanation of Experimental results are indicated in section “Comparison of detection rate using MIPM, IPM and Homography methods in public datasets”.

### Inherent geometry characteristic

**Fig 8:**
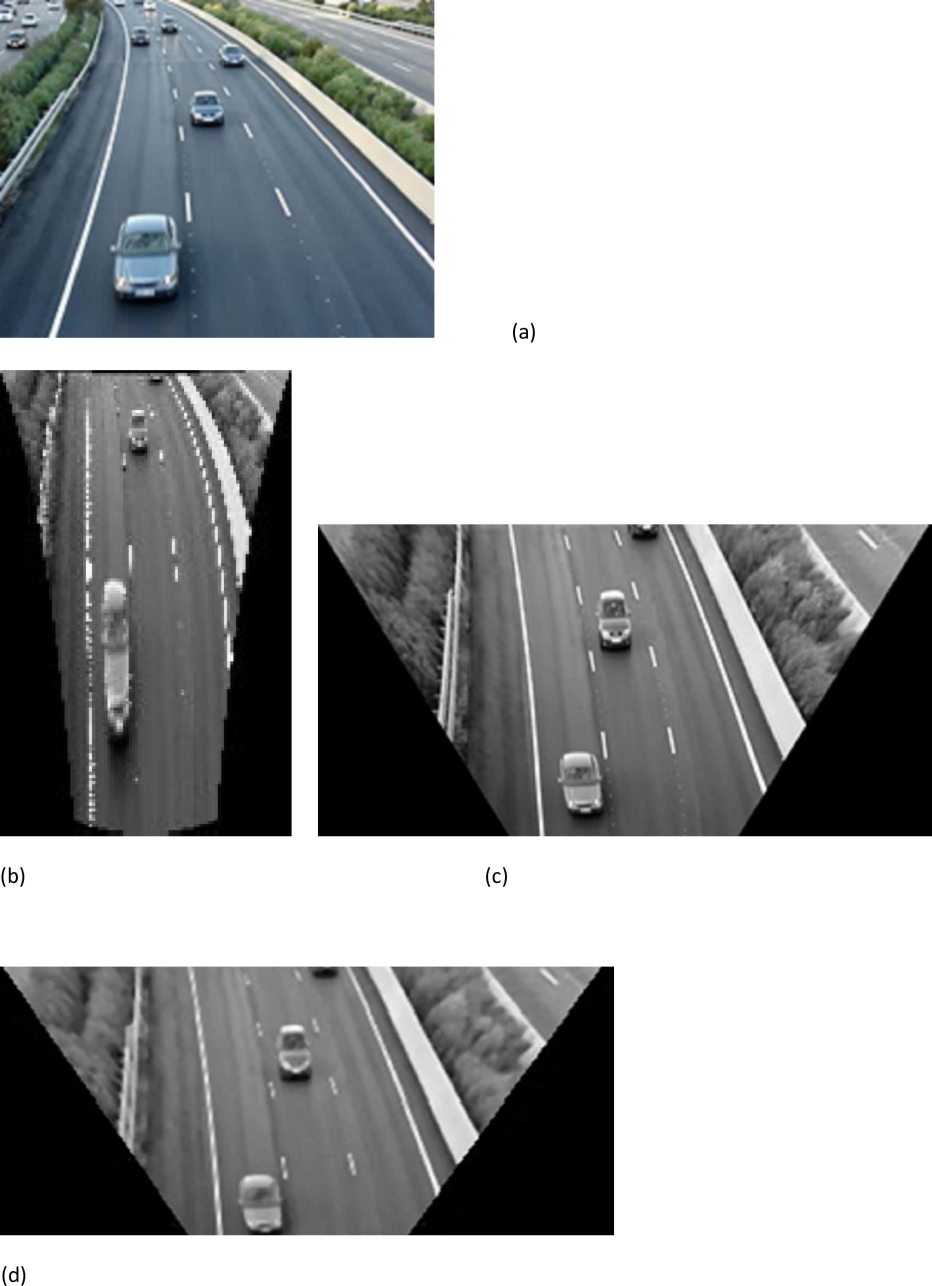
a) Original image with perspective effect b) Remapped image that removed perspective with IPM c) Remapped image that removed perspective with MIPM d) Remapped image with Homography method.

To obtain real information from the original images taken by a traffic camera, it is better to remove perspective from images. To do so, we used the previously commented method called Modified Inverse Perspective Mapping (MIPM). When using MIPM to remap images, geometric features of the road can be easier and more efficiently extracted from the remapped picture compared to the IPM and Homography version “Fig 8”.

### Locating the lane area with Hough transform

It is required to locate lane areas so that we become able to detect a vehicle queue in the video frames. In this work, we used Hough transform due to its structure, and its ability to consider locations and detect straight lines which were colored white. The accuracy for line detection is overlap with ground truth. Marking the individual lane marker locations in images is a common approach to generate ground truth. In this paper, to extract local maxima or bright points, from the accumulator array we used thresholding, then we applied some thinning to the isolated clusters of bright points in the accumulator array image. We took those local maxima whose values were equal or greater than some fixed percentage of the global maximum value. An accumulator covering the hough space is used to determine the areas where most Hough space lines intersect.

**Fig 9:**
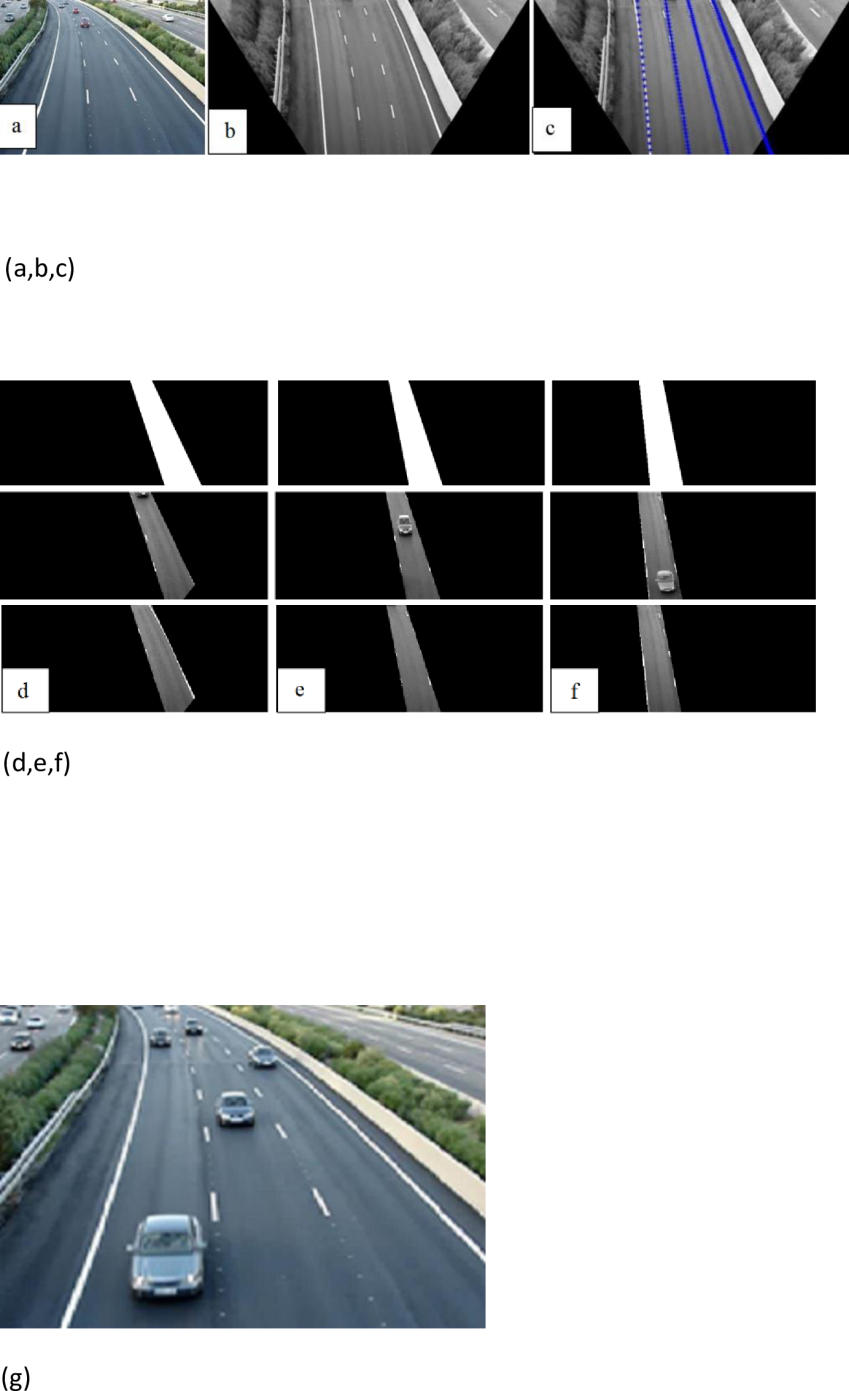
(a-g) a) Original image b) Removed perspective with MIPM c) Detected lines d) lane1 e) lane2 f) lane3 g) Original image.

In the sample image, no vehicle was included “Fig 9”. Actually, in this figure, lines are not straight, but Hough transform can be applied for curve detection if we know about the location of a boundary, in which its shape can be described as a parametric curve (e.g., a straight line or conic). We know the results will not be affected by gaps in curves and by the noise. Road lines were chosen to define lane areas “Fig 9”.

### Detection of vehicles

**Fig 10:**
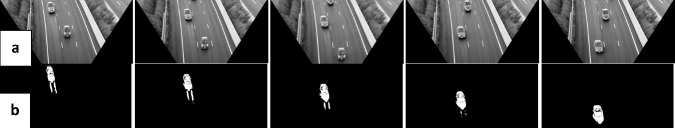
Detection of vehicle in lane 1 of the high way a) Remapped by MIPM b) Detected vehicle in lane 1 by GMM.

“Fig 10” shows detection of vehicles in lane 1 of the highway. Test results indicate that, using the presented methods, one can extract real properties of the vehicles such as area, width, length as well as the distance between the vehicles. However, to improve the results, it is much better to remove noise from images, such as the shadow detected by GMM.

### Removing of shadows in HSI space

Our proposed method works based on a modified version, (There are many published methods, used HSV color space). The chromacity information was applied to create a mask of candidate shadow pixels, followed by the gradient information to remove foreground pixels that were incorrectly included in the mask. In our work, to remove shadows with the chromacity-based method, first, space color of the images should be converted from RGB to HSI, because the intensity differentiation of shadow and object region is better visualized in HSI color space than RGB, HSV, etc[43].

**Fig 11:**
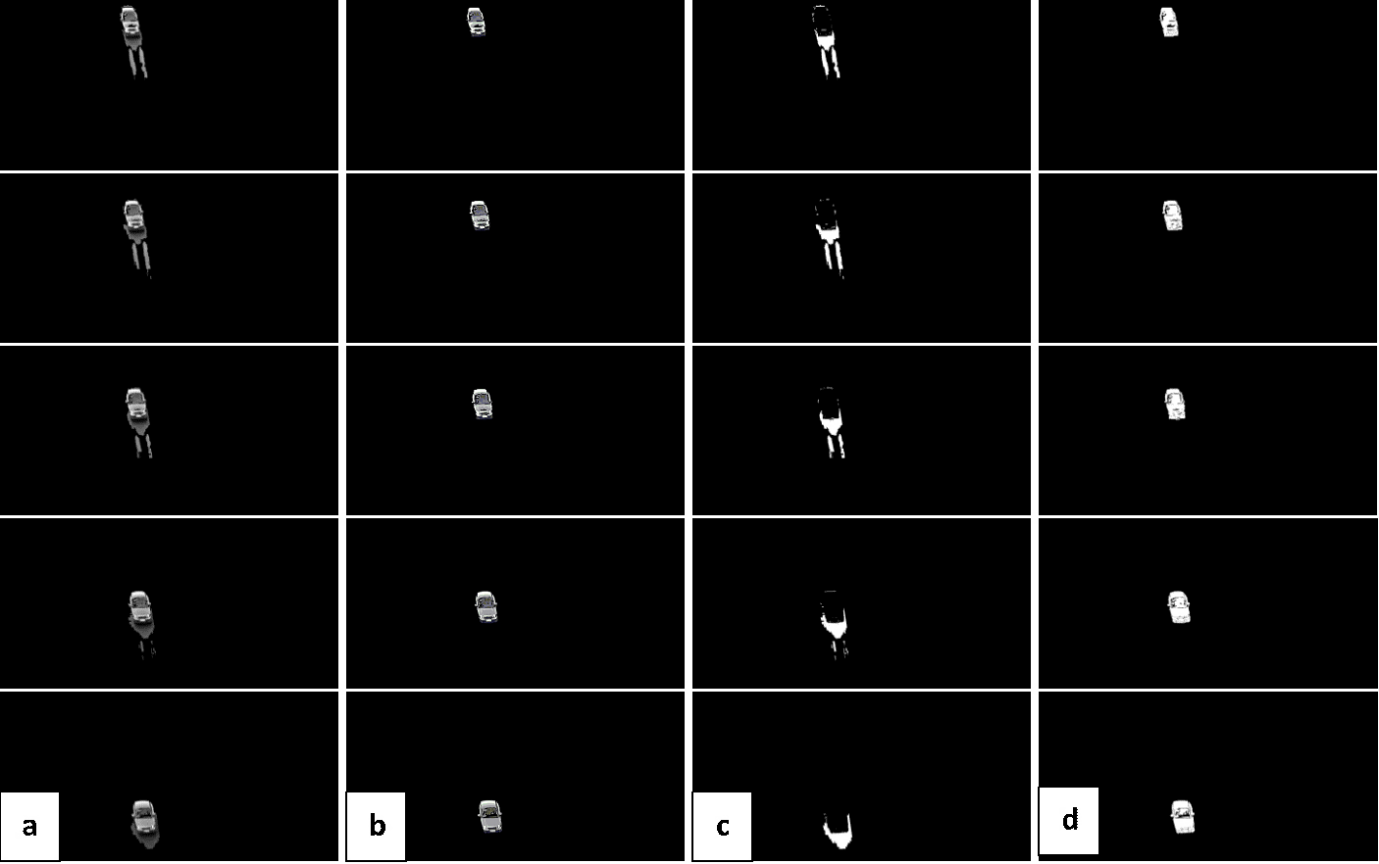
Removal of shadows with the chromacity-based method in HSI color space a) Results of dot product between original mages and binary images b) Removal of shadows in the original images c) Shadows are detected d) Results of the difference between the shadow images and the binary ones.

Using Eqs 12, 13 and 14 and differences between the shadow and the vehicle in the images, we extract boundary for hue, saturation and intensity “Fig 11”.

#### Quantitative results of shadow removal

Prati et al. [44] have evaluated shadow detection methods using the following two metrics, which indicate the shadow detection rate(η) and the shadow discrimination rate (ε).

$$\eta =\frac{TP_{S}}{TP_{S}+FN_{S}}.$$ (15)

$$\varepsilon =\frac{TP_{F}}{TP_{F}+FN_{F}}.$$ (16)

Here TP and FN are true positive and false negative pixels with respect to either shadows (S) or foreground, objects (F). The shadow detection rate is concerned with labelling the maximum number of cast shadow pixels as shadows. The shadow discrimination rate is concerned with maintaining the pixels that belong to the moving object as foreground.

The values of the shadow detection rate (η), shadow discrimination rate (ε) and time per frame are presented in “Table 1”. The averages below were obtained in 45 labelled sequences in different weather conditions and locations. The results show “Table 1” that, the method is able to achieve both high detection and discrimination rates even in bad conditions.

**Table 1:** Comparison of shadow detection methods.

Methods	(ƞ)%	(ε)%	Time (ms)
Chromacity Based Method (HSI space color)	97.58	98.72	7.21
Gradients	75.93	83.64	10.48
Additive shadow removal	82.10	79.54	11.92

### Detection and tracking of the position of the vehicles on the road

**Fig 12:**
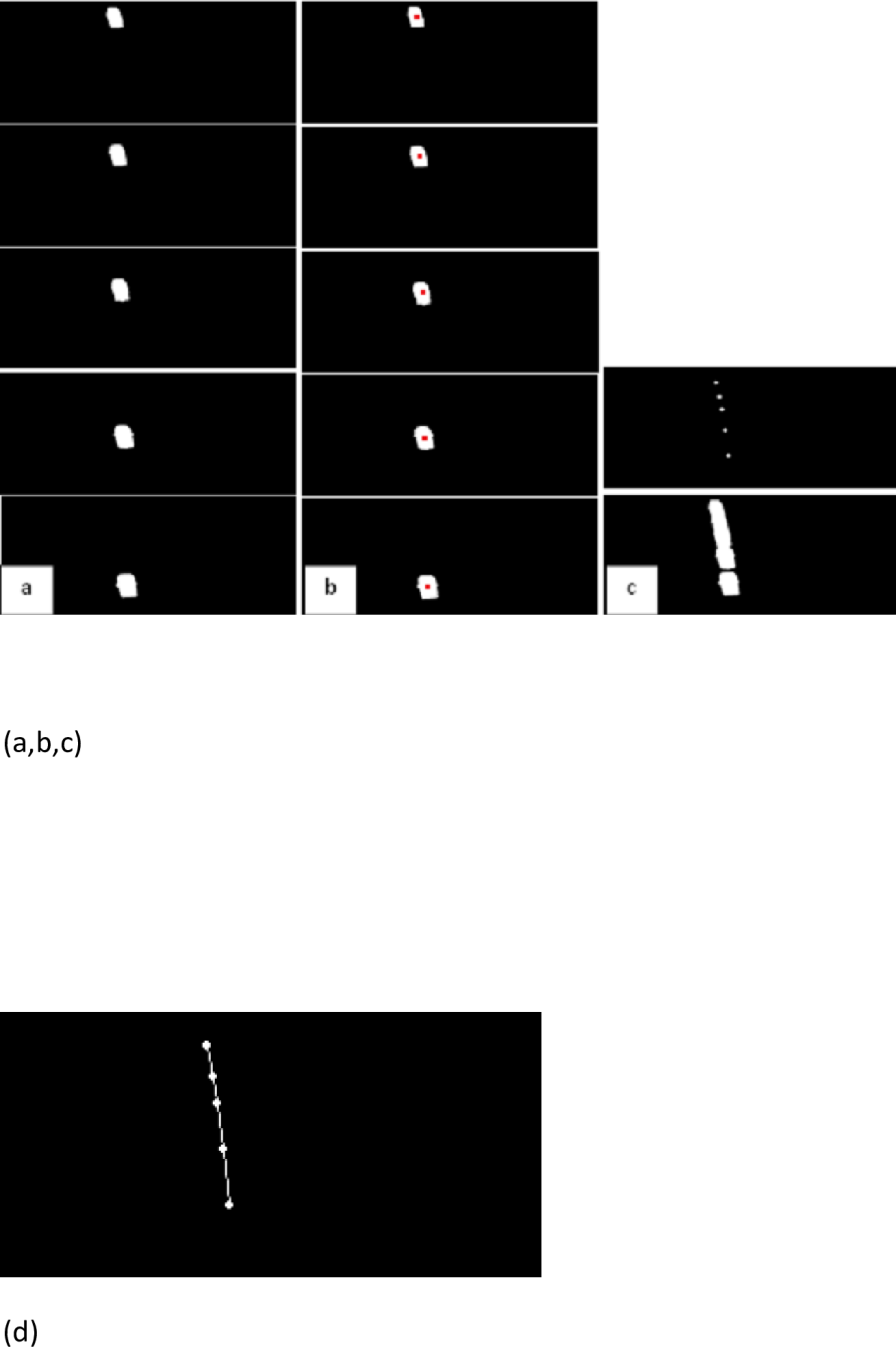
a) Binary images b) Geometric centers (red points) c) Aligning the geometric centers (white points) d) Path of vehicle (path tracking by frame-by-frame evaluation of blobs centers).

To detect the position of a certain vehicle on the road, first, all objects of the images were labeled; then, the geometric center of each one of the vehicles was determined. Each geometric center is considered as one vehicle “Fig 12”. Moreover, the path of a vehicle can be obtained by aligning its geometric centers in consecutive frames “Fig 12”.

## Comparison of the different methods and validation

**Fig 13:**
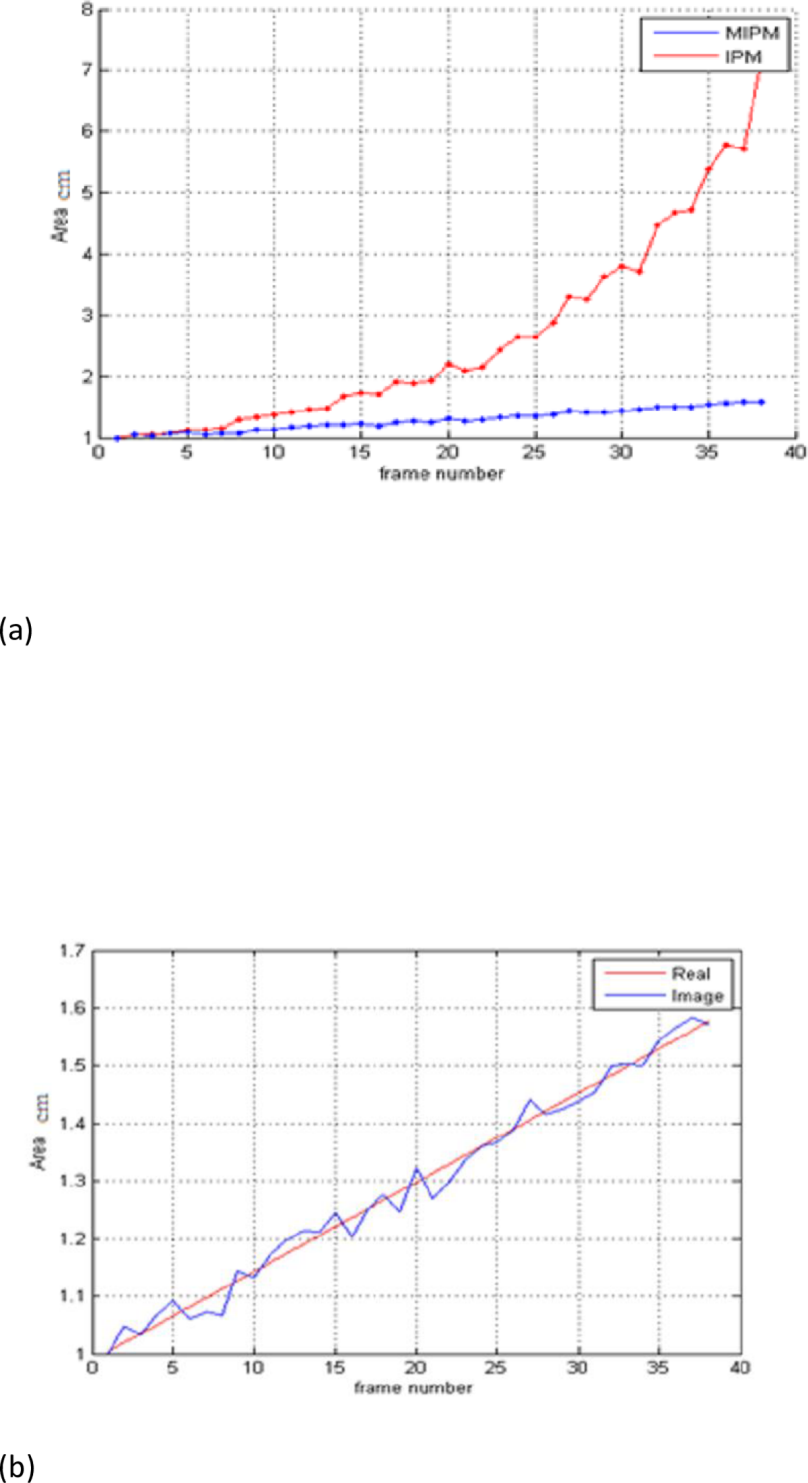
(a) Car area variation along with its direction using the two methods: IPM (red) and MIPM (blue) (b) Comparison of actual vehicle’s area and obtained area from image considering position and direction of the vehicle. Results indicate that areas measured using the presented MIPM method are closer to the actual ones in the 90% of the tested cars (compared to real areas with R^2^>0.98).

We tested our proposal against several kinds of traffic sequences, including low traffic, high traffic, and different locations (highway, urban, interurban and intersection). In addition, we tested different lighting conditions (sunny, poor lighting), and different weather conditions (snowy, rainy, normal). Also, different testing sequences that contained different types of cars (small and large vehicles) were selected to test our method. Below, the performance of the proposed MIPM is compared with that of the conventional IPM and Homography method. For this purpose, first, different types of cars in different locations and conditions were randomly selected. We applied MIPM, IPM and Homography methods to the images. Then, using Gaussian Mixture Model and Chromacity-based Method we segmented the cars, removed the shadows, and finally the vehicles areas were calculated. This was used as a measure to evaluate the performance of IPM, homography and MIPM. “Table 2” and “Fig 13” show the obtained results related to a certain vehicle. So, Eq 17 is used to normalize the vehicle’s area for different sizes of the vehicles. In other words, the area of the vehicle is compared to the first frame.

**Table 2:** Car area variation along with its direction using the 3 methods, IPM, Homography and MIPM.

N Frame	1	2	3	4	5	6	7	8	9	10	11	12	13	14
MIPM	1	1.04	1.03	1.06	1.09	1.06	1.07	1.06	1.14	1.13	1.17	1.19	1.21	1.21
IPM	1	1.04	1.05	1.06	1.12	1.13	1.14	1.30	1.34	1.34	1.41	1.46	1.47	1.66
Homography	1	1.04	1.05	1.07	1.13	1.12	1.14	1.30	1.36	1.34	1.42	1.48	1.51	1.60
N Frame	15	16	17	18	19	20	21	22	23	24	25	26	27	28
MIPM	1.24	1.20	1.25	1.27	1.24	1.32	1.26	1.29	1.33	1.36	1.36	1.38	1.44	1.41
IPM	1.72	1.69	1.90	1.89	1.92	2.19	2.10	2.14	2.44	2.65	2.64	2.87	3.29	3.25
Homography	1.77	1.71	1.85	1.86	1.91	2.23	2.19	2.19	2.31	2.57	2.65	2.71	3.35	3.47
N Frame	29	30	31	32	33	34	35	36	37	38				
MIPM	1.42	1.43	1.45	1.49	1.50	1.50	1.54	1.56	1.58	1.57				
IPM	3.62	3.80	3.70	4.46	4.67	4.70	5.38	5.77	5.71	7.12				
Homography	3.64	3.71	3.71	4.22	4.52	4.79	4.79	5.81	5.80	6.52				

$$\text{Area Normalization}=\frac{\text{area of vehicle}}{\text{Base area of vehicle}}.$$ (17)

“Table 2” show the computed area of the vehicles in increasing frames, using MIPM, IPM and Homography (our MIPM has better result with more accuracy).

### Highlighted advantages of the proposed method against IPM and homography

Variations of car areas along with their directions,“Fig 13A” and “Table 2” illustrate that MIPM is not only able to successfully remove perspective with a suitable scale, it can also create better estimations for the moving objects. Accordingly, it can be inferred that MIPM outperforms IPM and Homography “Table 2” as when we use MIPM:

- The area of the vehicle is constant in each location.
- The measured distances between vehicles are more accurate.
- The measured distances between the vehicle and camera are more accurate.
- The measured width of the road and the vehicle is more accurate.

To further evaluate the performance of IPM, homography and MIPM, the measured areas of different cars in different locations were compared with the reported areas by the manufacturing companies (ground truth). Accordingly, every square millimeter was considered as a pixel, then, Eq 17 was applied to normalize the results. Considering the position and direction of the vehicles, we measured the vehicle’s area in the images and compared them with actual areas. The results relating to one of the vehicles are illustrated in “Fig 13BB”.

**Fig 14:**
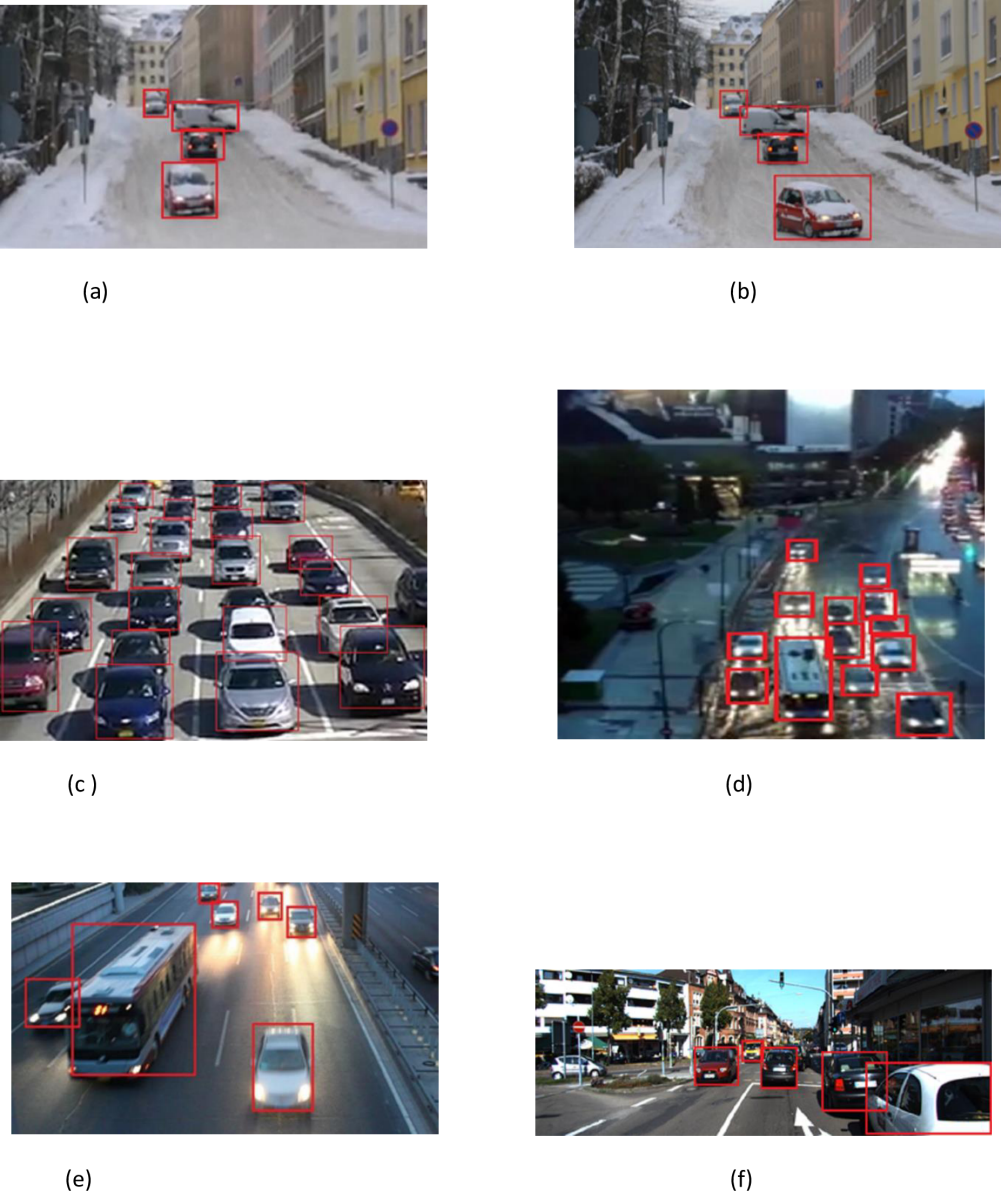
Some sequences in different weather conditions and locations and result of our detection and tracking using our MIPM method (a-f). (a) Urban area, snowy weather with strong wind, frame n 56 (our dataset), b) Urban area, Snowy weather with strong wind, frame n 69 (our dataset), (c) Sunny weather with high traffic at high way frame n 411 (our dataset), (d) Urban area, high traffic, poor lighting in rainy weather, frame n 802 (our dataset), (e) High way, light traffic, poor lighting in normal weather, frame n 1494 (DETRAC dataset), (f) Intersection, sunny day, day traffic, Frame n 61 (KITTI dataset).

As shown in the “Tables 2 and 3” and “Figs 3, 8, 9, 10, 13 and 14”, MIPM performance is comparable with IPM and Homography method in the first frames, but it is different in the following ones.

**Table 3:** Comparison of detection rate using 3 Methods on KITTI and DETRAC datasets.

KITTI				DETRAC		
Method	P1 Urban %	P2 Urban Two-way road %	P3 Urban Multi-lane road %	P4 Cloudy day %	P5 Sunny day with day traffic %	P6 Poor lighting condition %
Homography	96.82	96.50	97.71	97.53	98.36	89.94
IPM	97.34	98.11	98.59	97.15	98.83	87.29
Proposed Method MIPM	99.57	99.38	98.91	99.26	99.75	96.48
Ground truth	980	1289	1328	1022	1307	853

### Comparison of detection rate using MIPM, IPM and Homography methods in public datasets

We used MIPM, IPM and homography for removing the perspective effect in our system with the well-known KITTI and DETRAC datasets. The result show “Table 3” that our MIPM method is able to increase the detection rate.

We randomly selected 50 frames for each part. Note that number of vehicles were counted manually (Ground Truth).

The comparative evaluation of “Table 3” is based on the same metric (Detection Rate—DR), and the same evaluation criteria were applied to MIPM, IPM and homography.

True Positive Rate or Detection Rate (DR) or Recall:

$$DR=\frac{TP}{TP+FN}\times 100.$$ (18)

## Detection using MIPM method

In “Table 4” videos vary by several factors as location, occlusion, weather conditions, shadow, illumination (different hours in 6 days), different traffic volume, road dimension, different type of vehicles, camera view angle and region of view. Vehicle surveillance systems undergo various difficulties especially in urban traffic scenarios, such as road sections and intersections in which dense traffic, vehicle occlusion and orientation variation highly affect their performance [45].

**Table 4:** Analysis of detection rate of our method (MIPM), with 3 different data sets.

Data set	Testing sequences	Total number of vehicles	Number of detected vehicles	Vehicle detection (Correct Rate) %	Lane detection %	Shadow removal with vehicle detection %	Success Rate %	False Rate %
Low Traffic	30	172	171	99.41	99.70	99.40	99.50	1.52
High Traffic	30	647	635	98.20	99.20	98.71	98.70	2.97
Highway	30	510	507	99.41	99.90	99.60	99.63	1.52
Urban	30	436	434	99.54	98.91	99.70	99.38	1.18
Interurban	30	371	368	99.19	99.30	99.32	99.26	1.69
Intersection	30	205	200	97.56	98.10	98.50	98.05	4.81
Poor lighting condition	30	164	158	96.34	99.32	97.12	97.58	6.24
Snowy Weather	30	196	193	98.46	96.81	98.92	98.05	2.73
Rainy Weather	30	292	269	92.12	97.92	93.91	94.64	8.59

Table 4 was evaluated for counting vehicles. It shows the number of detected cars by our method and the total number of cars (manual count). Additionally, an analysis of the correct rate (correct detected vehicle) and success rate are considered. For this purpose, our method was used several times, then the best success rates were considered as table values. According to Table 4, the average rate of vehicle detection in different areas and conditions is 97.80%, and lane detection rises to 98.78%. Shadow removal is good enough to provide vehicle detection with an average rate of 98.34%.

Additionally, we used false positive and correct rate to obtain car and lane detection for our comparison. For this purpose, pixel wise evaluation is used to compute these parameters. The frame rate of the proposed method is 10 fps. We selected sequences randomly from our database, KITTI and DETRAC datasets for this test. False positive should be understood as vehicles detected by our system, but actually not present in the ground truth.

FPR: It is a measure of how well the system correctly rejects false positives, and is defined as:

$$\text{False Positive Rate}=\frac{\text{FP}}{\text{FP}+\text{TN}}\times 100.$$ (19)

The global Success Rate is computed as the success rate of vehicle detection, plus success rate of the shadow removal module.

$$\text{True Positive Rate}=\frac{\text{TP}}{\text{TP}+\text{FN}}\times 100.$$ (20)

The proposed method is time efficient and can be used for real time applications like counting the number of vehicles.

The detection results from different data sets show that our method is quite effective. The occlusions of cars in the video sequences are comparable to those in the static image set. Note that the detection rate on video is computed in a ground-truth way. We labeled the vehicles manually, then we compared the detection results and the labeled data of every frame to compute the detection rate as Success Rate. “Fig 14” shows some sequences in different weather conditions, locations and results of our detection and tracking system using MIPM. “Fig 14A and 14B” shows simultaneous tracking of vehicles, including snowy weather with strong wind.

Considering our algorithm, it is less time consuming and has a higher detection rate, outperforming others, as indicated in “Tables 5–9.”

**Table 5:** Selected sequences are in urban area with sunny weather.

	Ground truth (number of vehicles)	Detected vehicles	Detection rate %	Accuracy %	Frame rate (Frame/sec)
Our Method	1100	1097	99.72	99.72	10
GMM and Kalman filter [17]	1100	1077	97.9	95.61	7
GMM and trained images with optical flow [18]	1100	1024	93.09	94.12	5

**Table 6:** Poor lighting conditions in highway.

	Ground truth (number of vehicles)	Detected vehicles	Detection rate %	Accuracy %	Frame rate (Frame/sec)
Our Method	614	597	97.23	98.90	10
GMM and Kalman filter [17]	614	502	81.75	83.23	7
GMM and trained images with optical flow [18]	614	519	84.52	85.10	5

**Table 7:** In urban area with snowy weather.

	Ground truth (number of vehicles)	Detected vehicles	Detection rate %	Accuracy %	Frame rate (Frame/sec)
Our Method	293	289	98.63	98.70	10
GMM and Kalman filter [17]	293	231	78.83	75.10	7
GMM and trained images with optical flow [18]	293	252	86	87.91	5

**Table 8:** Rainy weather in highway.

	Ground truth (number of vehicles)	Detected vehicles	Detection rate %	Accuracy %	Frame rate (Frame/sec)
Our Method	201	189	94.02	96.70	10
GMM and Kalman filter [17]	201	185	92.03	95.73	7
GMM and trained images with optical flow [18]	201	166	82.58	81.34	5

**Table 9:** High traffic in an interurban (with occlusion).

	Ground truth (number of vehicles)	Detected vehicles	Detection rate %	Accuracy %	Frame rate (Frame/sec)
Our Method	780	777	99.61	98.90	10
GMM and Kalman filter [17]	780	725	92.94	96.10	7
GMM and trained images with optical flow [18]	780	691	88.58	85.32	5

We are able to detect and simultaneously track “Fig 14” the set of vehicles present in the monitored area in different conditions, which may increase to approximately 26. Our simulation results confirmed that our method (MIPM) provided the most accurate vehicle detection results.

### Comparison to other state of the art methods

We compared our robust detection method (using MIPM) with other methods of the state of the art, “Tables 5–9.”We selected 50 sequences randomly from 3 different datasets in urban areas with sunny weather “Table 5”, then we tested our method with methods used in [17] and [18]. For ground truth, we manually counted vehicles within the frames. The results indicated that our method provides a higher detection rate compared to others of the state of the art We selected 30 frames randomly “Table 6” in highway with poor lighting conditions, to challenge our method. The results show that our method provides again better detection rates under poor lighting conditions, reaching the 97.23%.

We selected 30 frames randomly “Table 7” in urban areas with snowy weather and 30 frames in rainy weather “Table 8” to compare our method, obtaining 98.63% and 94.02% respectively. Finally, we also selected 30 frames randomly in high traffic in interurban areas, obtaining the good results shown in “Table 9”.

The performance of our method is measured using the Receiver Operating Characteristic (ROC) analysis. The parameters in the ROC analysis are:

TP (True Positive) or Correct Detection: The number of correctly detected true vehicles.

FN (False Negative): The number of vehicles that are not detected.

FP (False Positive): The number of the vehicles present in the system under test, but not in the GT (Ground truth).

TN (True Negative): True negative, the vehicles present in neither the GT nor the system under test.

True Positive Rate or Detection Rate (DR) or Recall is $DR=\frac{TP}{TP+FN}\times 100$ and accuracy can be defined as $Accurancy=\frac{TP+TN}{TP+FN+FP+TN}\times 100.$

Also *the total number of the ground truth vehicles* = *TP* + *FN* + *FP* + *TN*.

In our method, the testing sequences are eliminated as follows: 1) too many occlusions in the image (vehicles occluded by a front larger vehicle) 2) the majority of vehicles in the image are not front-view 3) the resolution of the image is too low, e.g., the image is blurred or the vehicles are too far from our camera. Using an image-label tool, we created positive samples from these images. The diversity of occlusions over the positive samples are noticeable, i.e., the positive samples with different occlusion situations should be included in the training set and the numbers of samples representing each situation are almost identical to the true distribution of this situation in the real world. However, it’s difficult to obtain this information. So, in our method, the numbers of each occlusion are set equally. We treat the occlusion situations as three types for cars: 1) two or three successive cars occlude one by one in the same lane 2) one car is behind a car in the same lane 3) one car is occluded by the vehicle driven towards the same lane. The structural information, like lanes or traffic markings, are sometimes badly marked or even hard to identify in the presence of harsher weather conditions.

We observed that those vehicles were not detected for the following reasons; direct sunlight into the camera, white cars in snowy weather, vehicles were too far from the camera and occluded by larger vehicles, vehicles in an intersection and had no line of sight with one camera, and uphill/downhill roads made vehicle detection difficult. Here we have indicated the limitation of our system in the above mentioned points.

## Conclusions and future work

In this research, we have proposed a robust method for extracting real information from traffic cameras. The research focused on different issues, namely removing perspective, automatic locating of lines and lanes, vehicle detection and extracting features of the vehicles. As the main contribution of this research, we have proposed a method to remove perspective without any harmful effect on the real information. Experimental results indicate that the proposed method, called Modified Inverse Perspective Mapping (MIPM), was not only simple and straightforward, but it was also more accurate compared to the state-of-the-art methods. However, the proposed method was not tested under camera vibrations and dusty weather. From the results obtained in the previous sections, despite having the same information, the proposed MIPM method is of better clarity and transparency compared with the homography, IPM and other methods.

Therefore, in our future studies, we will work on generalizing the proposed framework to become robust against conditions such as dusty weather and bad lighting specifically at night. We will propose a method for high detection rates of vehicles in tunnels, winding roads and steep uphill roads. Moreover, to generalize our framework to 3D information extraction, we are planning to use two cameras instead of one to remap 2D space to 3D.

## Supporting information

S1 File Some examples of tested data set in Madrid and Tehran. (DOCX)
