# Supplementary material for: Robust vehicle detection in different weather conditions: Using MIPM
Source: PLoS One. 2018 Mar 7;13(3):e0191355. doi: 10.1371/journal.pone.0191355 (PMC5841654; doi:10.1371/journal.pone.0191355)
Supplement: S1 File — (DOCX) [file pone.0191355.s001.docx]

We have used three datasets.

- Two well-known public datasets KITTI and DETRAC. They can be find at:

41. The KITTI Vision Benchmark Suite. [Online].

Available: http://www.cvlibs.net/datasets/kitti/ (current October 2017)

42. The UA-DETRAC Benchmark Suite. [Online].

Available: http://detrac-db.rit.albany.edu/ (current October 2017).

- Also, our captured dataset in Madrid and Tehran.

These videos were captured under normal, sunny, snowy, rainy weather conditions at different urban, interurban, intersection environments and high-ways in Madrid and Tehran. They contain different types of locations, different lighting conditions and occlusions between cars. We collected more than 80 videos, some of them up to 5 minutes in length. Over a period of more than six days for different conditions.

Our simulation results indicate that the proposed algorithms and frameworks are effective, robust, and more accurate compared to other frameworks, especially when facing different kinds of occlusions, varying in lighting conditions, weather conditions and environments. More details about datasets can be find in section 3 and comparison with other results in section 4. Please note that location, weather conditions and the date of each videos are in the links. Some links include more than 1 videos.

(due to strong wind and bad weather conditions some videos are unclear.)

<https://figshare.com/s/d4e7ba0d301e5abf698f>

<https://figshare.com/s/cdcbfbecb3668426b7f3>

<https://figshare.com/s/34326362399d57091c1a>

<https://figshare.com/s/836294062a0e463ca110>

<https://figshare.com/s/b5dbfc20c20e23ff8b7c>

<https://figshare.com/s/f44254378ae258556999>

<https://figshare.com/s/02abd48aabe53f8aa749>

<https://figshare.com/s/c6dc85bd6b1839e63c53>

<https://figshare.com/s/f7467d15e3a1f4cf9c9f>

<https://figshare.com/s/1d19d834801fa6275801>

<https://figshare.com/s/be48e75f69118e8b7f0c>

<https://figshare.com/s/ba5b70157e80f93b2d73>

<https://figshare.com/s/fd461fccf23a3b6a3a0a>

<https://figshare.com/s/97b8850b9f6c253c5080>

<https://figshare.com/s/c162f46cf72281760a13>

<https://figshare.com/s/537ed977a33ed606d006>

<https://figshare.com/s/7ab9a017b2d0b6362c1b>

<https://figshare.com/s/14f08c6b06a6626334e8>

<https://figshare.com/s/4ab1fb67259de4a5c1a9>

<https://figshare.com/s/a2027764c808304e1672>

<https://figshare.com/s/04fa8c83f435bc6ca39d>

<https://figshare.com/s/782eed50118998e9dcb3>

<https://figshare.com/s/e08216e1581f38713ce6>

<https://figshare.com/s/f771f9bf116f595f934c>

<https://figshare.com/s/a6ac2269ab459439b4b4>

<https://figshare.com/s/e658a6e5d2ecfa23dacd>

<https://figshare.com/s/c0fffb0126ca37d1d7fd>

<https://figshare.com/s/096f0293b8fe17fad7c5>

<https://figshare.com/s/44a9b7dd897498086669>

<https://figshare.com/s/aaa4b6a4d345faa9123d>

<https://figshare.com/s/7f4c78dc02a0c25070ec>

<https://figshare.com/s/bb5a8c06e747a3e2360c>

<https://figshare.com/s/995e0306f6a334b84032>

<https://figshare.com/s/5b4b51b508f952dfa4b5>
